# IoTCrawler: Challenges and Solutions for Searching the Internet of Things

**DOI:** 10.3390/s21051559

**Published:** 2021-02-24

**Authors:** Thorben Iggena, Eushay Bin Ilyas, Marten Fischer, Ralf Tönjes, Tarek Elsaleh, Roonak Rezvani, Narges Pourshahrokhi, Stefan Bischof, Andreas Fernbach, Josiane Xavier Parreira, Patrik Schneider, Pavel Smirnov, Martin Strohbach, Hien Truong, Aurora González-Vidal, Antonio F. Skarmeta, Parwinder Singh, Michail J. Beliatis, Mirko Presser, Juan A. Martinez, Pedro Gonzalez-Gil, Marianne Krogbæk, Sebastian Holmgård Christophersen

**Affiliations:** 1 Faculty of Engineering and Computer Science, University of Applied Sciences Osnabrück, 49076 Osnabrück, Germany; e.bin-ilyas@hs-osnabrueck.de (E.B.I.); m.fischer@hs-osnabrueck.de (M.F.); r.toenjes@hs-osnabrueck.de (R.T.); 2 Centre for Vision, Speech and Signal Processing, University of Surrey, Guildford GU2 7XH, UK; t.elsaleh@surrey.ac.uk (T.E.); r.rezvani@surrey.ac.uk (R.R.); n.pourshahrokhi@surrey.ac.uk (N.P.); 3 Siemens AG Austria, 1210 Vienna, Austria; bischof.stefan@siemens.com (S.B.); andreas.fernbach@siemens.com (A.F.); josiane.parreira@siemens.com (J.X.P.);; 4 AGT International, 64295 Darmstadt, Germany; PSmirnov@agtinternational.com (P.S.); MStrohbach@agtinternational.com (M.S.); 5 NEC Labs Europe, 69115 Heidelberg, Germany; hien.truong@neclab.eu; 6 Information and Communication Engineering Department, University of Murcia, 30100 Murcia, Spain; aurora.gonzalez2@um.es (A.G.-V.); skarmeta@um.es (A.F.S.); pedrog@um.es (P.G.-G.); 7 Department of Business Development and Technology, Aarhus University, 7400 Herning, Denmark; parwinder@btech.au.dk (P.S.); mibel@btech.au.dk (M.J.B.); mirko.presser@btech.au.dk (M.P.); 8 Odin Solutions, R&D Department, 30820 Murcia, Spain; jamartinez@odins.es; 9 City of Aarhus, 8000 Aarhus, Denmark; mkrog@aarhus.dk (M.K.); sech@aarhus.dk (S.H.C.)

**Keywords:** Internet of Things, search, security, privacy, reliability, IoT search framework, IoT data sources

## Abstract

Due to the rapid development of the Internet of Things (IoT) and consequently, the availability of more and more IoT data sources, mechanisms for searching and integrating IoT data sources become essential to leverage all relevant data for improving processes and services. This paper presents the IoT search framework IoTCrawler. The IoTCrawler framework is not only another IoT framework, it is a system of systems which connects existing solutions to offer interoperability and to overcome data fragmentation. In addition to its domain-independent design, IoTCrawler features a layered approach, offering solutions for crawling, indexing and searching IoT data sources, while ensuring privacy and security, adaptivity and reliability. The concept is proven by addressing a list of requirements defined for searching the IoT and an extensive evaluation. In addition, real world use cases showcase the applicability of the framework and provide examples of how it can be instantiated for new scenarios.

## 1. Introduction

During the last years, the Internet of Things (IoT) has grown massively and is still growing because of the availability of cheap sensor devices and more and more widespread IoT frameworks, increasing the number of devices and services. This leads to new possibilities for use cases and scenarios in the IoT (e.g., http://www.ict-citypulse.eu/scenarios/ accessed on 24 February 2021). These scenarios range from agriculture, Industry 4.0, to smart cities and many others. A quite common problem for all of these domains is the search and discovery of available IoT devices, which is the main purpose of the IoTCrawler framework.

To realize the envisaged IoT search framework, a two-layered approach is foreseen, containing the Discovery and Processing Layer and the Search and Orchestration Layer. The term Discovery refers to the process of connecting new data sources to the framework. This may require a step to extract additional information from other databases named Crawling. The Processing refers to actions to ease up and enhance the later search. Processing includes the Indexing, i.e., preparing ordered references to discovered data sources for faster access; the Semantic Enrichment (SE), i.e., the deduction of new data, either describing higher-level context or the data stream itself. The Search and Orchestration Layer becomes active when a search process is started. Search refers to the act of finding suitable data sources in the system by an application and includes a ranking mechanism to sort out the results to fit best the specific use case. Creating the ability for an application to receive live observations from a data stream is done during the orchestration step. When designing an IoT search framework, there are several issues to be considered: volume (the amount of data), heterogeneity (different kinds of data sources), dynamics (changes in data sources/environments) and security and privacy (e.g., IoT data sources measuring sensitive data). By analysing these issues, a number of general requirements for an IoT search platform can be derived:

**R-1**  **Scalability:** Coming from the issue of Volume, a requirement for scalability arises when designing products for the IoT. The huge amount of available, and often heterogeneous, data sources, which have to be considered for the process of search, leads to a challenge of scalability. All components and solutions in this environment have to be designed to work with large scale data. As a result, the machine initiated search shall be answered within a reasonable time.
**R-2**  **Semantics and Context for Machine Initiated Search:** Newly emerging search models require to tackle the search problems based on the human- and machine originated users’ contexts and requirements such as location, time, activity, previous records and profile. The search results are targeted to be based on emerging IoT application models, where search can be initiated without human involvement. The generation of higher-level context, such as traffic conditions, e.g., from low-level observations, can enhance the search functionality for applications that require information on trends and profiles about sensory data. Generated data from IoT deployments are largely multivariate, and therefore require aggregation methods that can preserve and represent its key characteristics, while reducing the processing time and storage necessities.
**R-3**  **Discovery and Search:** To provide a well performing and responsive IoT search framework, the entire process needs to be considered as a two stages approach, namely Discovery and Search. In the first stage, knowledge about available IoT devices and the data streams they provide has to be crawled. The goal is to build up a data repository containing available information about the data streams. In the second stage, while processing a search request, the potential data streams, satisfying the search query, are then extracted from the repository. Before being returned to the requester, the list of candidates needs to be ranked, to allow the application to use the best fitting data streams.
**R-4**  **Security and Privacy by Design:** It is vital that Privacy and Security are addressed from the beginning in a design phase and through all the development of a project. It requires authentication, access control and privacy mechanisms in order to provide a controlled environment where providers can specify the access policy attached to their data, and even broadcast it in a privacy preserving manner, so that only legitimate consumers are able to access the information.

While traditional IoT middleware platforms allow users to search for particular IoT devices, they still require manual interaction to integrate data sources into a use case. As the number of IoT devices has increased profoundly in last couple of years, many middlewares have also surfaced to introduce more flexibility and functionality to IoT solution providers. Middlewares like Kaa (https://www.kaaproject.org/ accessed on 24 February 2021) and SiteWhere (https://sitewhere.io/ accessed on 24 February 2021) provide features like data storage, data analysis, device management along with the tools to analyse infrastructure and optimise computation or provide additional functionality like digital twins in Kaa. MainFlux (https://www.mainflux.com/ accessed on 24 February 2021) and OpenRemote (https://openremote.io/ accessed on 24 February 2021) employ protocol and device agnostic strategies to ease the connectivity of devices. Distributed Services Architecture (DSA) (http://iot-dsa.org/ accessed on 24 February 2021) provide solutions for the devices to communicate in a decentralised manner. With all these different middlewares, there is still a lack of searching mechanisms that facilitates both Machine-to-Machine (M2M) and Machine-to-Human (M2H) communication. The main goal of IoTCrawler is to provide tools that answer search queries according to user’s preferences such as sensor types, location, data quality. For better M2M communication, automated context dependent access is provided based on a machine initiated semantic search. IoTCrawler also monitors these IoT devices and informs the users about changes in data quality and the availability of new relevant sensors to provide flexibility and additional information. Moreover, IoTCrawler envisions a platform which can provide any user an easy access to open data while also facilitating private users such as industries and businesses. For this, research has been conducted to implement strategies which ensure that private data stays protected and is only provided to the authenticated user.

This paper provides an overview of the IoT search framework IoTCrawler, which is able to crawl IoT data sources and provides an interface to allow for human- as well as machine-initiated search requests. The IoTCrawler framework consists of a series of loosely coupled components and is thoroughly designed to address the identified requirements. The components are designed to be used individually or as a whole framework to allow the search for IoT data sources in a fast, stable and secure way.

The remainder of this paper is organised as follows. Section 2 presents related work in regarding the solutions and components of the IoTCrawler framework. Section 3 describes the idea of IoTCrawler as a search framework for data sources in the IoT, while Section 4 and Section 5 depict the two layered approach and present the enablers for the discovery and the enablers for the search layer in detail, including solutions to address the presented requirements. Section 6 provides an overall evaluation of several IoTCrawler framework instances running for certain use cases in real-world environments. Finally, Section 7 concludes the paper.

## 2. Related Work

The question of search and discovery in the internet is not new. The developed techniques range from the well-known and widely deployed Domain Name System (DNS), the Lightweight Directory Access Protocol (LDAP), to decentralised Distributed Hash Table (DHT). However, none of them address all the challenges in the IoT domain introduced in Section 1. This section highlights the current technical state of topics relevant for a search engine in the IoT.

### 2.1. Search over Discovered Metadata

A number of approaches for managing IoT metadata and performing a search over it can be found in literature. In the Dyser search engine [1], a query-based search mechanism is used for tracking the states of physical entities in real-time. Using a typical link-traversing approach, it performs crawling and maintains the actual state of dynamically changing metadata. Another service for semantic search and sensor discovery among the Web of Things (WoT) is DiscoWoT [2]. Using a RESTful approach, it enables the integration of WoT entities. The service is based on extensible discovery strategies. Along with that, it allows publishers to semantically annotate WoT sources. The Thingful engine (https://www.thingful.net/ accessed on 24 February 2021) uses a ranking algorithm over geographical indexed resources. A map-based Web UI is provided for verified sensors with locations. A contextual search allows to query sensors based on their type and location and nearby surroundings. A wrapping approach for integrating real-time data sources is applied in a platform called Linked Stream Middleware (LSM) [3], which uses Semantic Web technology for integrating real-time physical sensory data. For annotating and visualising data, the platform exposes a web UI with a SPARQL endpoint for querying. A predefined taxonomy includes location, physical context, accuracy and other metadata used for displaying types of sensing devices. SPARQL 1.1 with federation extension is used for federating queries from distributed endpoints [4]. WOTS2E [5]—a search engine for a Semantic WoT proposes a novel method for discovering WoT devices and services and semantically annotated data related to IoT/WoT. The engine relies on results of traditional search engines (e.g., Google), where it crawls Linked Data endpoints (SPARQL), which are semantically analysed. For the relevant endpoints, metadata will be extracted and stored in the service description repository, used later by IoT applications such as WoT index. In [6], authors analyse the state-of-art literature about IoT search engines and conclude that the most influencing (citing) contributions were done around 2010. This explains the fact that major references might look obsolete in 2021. Along with that, authors outlined two major functionalities performed by IoT search engines (content discovery and search over it), proposed a so-called meta-path methodology, identified 8 types of meta-paths and classified search mechanisms of existing IoT search engines. According to their classification (combinations of R, D, S, F), search mechanisms of IoTCrawler are able to consider the following assets: aspects of streams representatives (R) and stream observations (Dynamic Content, D), semantics of sensors and sensing devices (Representatives of IoT things possessing streams, again R). Due to the use of ontologies (IoTStream, Sosa) and extensible GraphQL-based querying mechanism [7], a submission of new information models is not a problem for the IoTCrawler metadata storage. For example, one of the crawling mechanisms [8] uses the DogOnt ontology [9] and enriches the Metadata Repository (MDR) and searches over it by the following assets: (a) types of sensors and sensing devices; (b) types of electrical appliances connected to energy-metering smart home sensors. Submission of new ontologies and extension of search mechanism with their facets share the same principles and would easily let IoTCrawler for cover functionality aspects (F) of IoT things. Considering that, we can conclude that the search mechanism of IoTCrawler covers the most of the proposed meta-path categories (except of microsensors level, S) and competes the search capabilities of engines belonging to them. Together with other capabilities (such as security and publish-subscribe, virtual sensors) IoTCrawler framework outperforms capabilities of pure search IoT search engines.

### 2.2. Semantics, Ontologies and Information Models for Interoperability

Over the past decade, a number of efforts have been made to define information models for IoT using ontologies and semantic annotations, although since these ontologies are developed by different entities, they are bound to be a diverge in semantics, since the IoT domain is quite broad in general. An important focus of IoTCrawler is the description of sensors and IoT data streams. Regarding sensors, one of the main initiatives made in this field is the W3C SSN ontology [10]. It defines an ontology for describing Sensors and Observations, but also expands to Systems, Deployments and Processes. SOSA [11] was created as an extension to SSN to simplify the ontology and to separate Sensors and Observations from other concepts that are deemed relevant for Sensor and Observations management. IoT-lite [12] was an effort to bind the core concepts of SSN with IoT concepts that were not covered by it, such as the concept of Service, but to support the scalability of annotations to IoT resources in a minimalist manner. The Stream Annotation Ontology (SAO) [13] is another effort which extends SSN to address sensor data streams. It employs a class taxonomy for stream analysis techniques, which is useful for high granularity. For this reason, the IoT-Stream [14] ontology was developed to serve the framework by carrying the principles that were adopted for IoT-lite to data streams, in the sense that stream annotations should be annotated as minimally as possible to support scale in the context of IoT data, but also to be flexible to increase the granularity of annotation as needed by the system.

### 2.3. Security and Privacy in IoT

Security and privacy cover different areas such as authentication, authorisation, integrity, as well as confidentiality to name a few. In the scope of IoT, Abomhara and Køien [15] identified three different core aspects: privacy for humans, confidentiality of business processes and third-party dependability. They also classified different attacks related to eavesdropping communications, which together with traffic analysis techniques allow attackers to identify information with special roles and activities in IoT devices and data. Nevertheless, they state that there are still open issues related to privacy in data collection, sharing and management, as described by Riahi et al. [16]. Another security aspect which has gained a lot of attention in both academia and industry, is the combination of authentication and identity management. This is widely acknowledged in the literature, such as the works of Mahalle et al. [17] or Bernal et al. [18], the latter associates the term privacy-preserving to identity management with the objective of representing not only users, but also devices or services. These aspects, together with the access control, have been also dealt in different EU research projects, such as Smartie, SocIoTal or CPaaS.io, where the integration of these technologies are also proved as an appropriate solution for different domains such as smart buildings or smart cities. These projects also propose the use of access control mechanisms based on eXtendible Access Control Markup Language (XACML) [19], even in a decentralised manner by using Attriute-Based Access Control (ABAC) [20], and to deal with privacy over the data by using encryption techniques based on attributes, such as Perez et al. [21] which composes an identity.

Hwang [22] also raises the well-known concern regarding the security threats related to IoT, for example the possibility to overwhelm a system by means of a few IoT attackers using Denial-of-Service (DoS)-based attacks [23]. The most remarkable point from this paper’s perspective is that, as Hwang states, a demand exists for security solutions capable of supporting multi-profile platforms with different security levels. On the other hand, Hernandez-Ramos et al. [24] address the issue of security and privacy from the point of view of the smart city. In this work, the necessity of having a mechanism for empowering citizens to manage their security and privacy by tools such as access control management, as well as decentralised data sharing, are addressed. This idea is endorsed also in another research work [25] where they describe a future data-driven society requiring a harmonised vision of cybersecurity.

### 2.4. Reliability in IoT

In the past, reliability in IoT has been handled by diverse techniques and solutions, from quality analysis to algorithms for fault detection and recovery, or replacement of faulty data sources. The term Quality of Information (QoI) determines the “fitness for use” of an information that is being processed [26]. It has been originally described as a quality indicator in the context of database systems [27], but has also been used in several frameworks for information processing. The authors of [28] proposed a framework for data translation and identity resolution for heterogeneous data sources including QoI. In comparison to other frameworks, their framework relies on linked data sets instead of real-time data. Other frameworks using QoI are shown in [29] for dealing with security in the context of healthcare including QoI or [30], which deals with streaming data that are stored into a database. For later analysis, they also store calculated QoI bundled to the data. A subscription system for data streams, which are selected on their data quality, is proposed in [31]. Puiu et al. [32] focused on real-time information processing with integrated semantic annotation [33] and QoI calculation for fault-recovery and event processing. Whereas all of these solutions integrate QoI and some of them provide real-time capabilities and semantics, they are bound to specific domains and none of these solutions are flexible enough to work as a decoupled solution supporting different IoT sensors.

As a result of the recent popularity of IoT, different platforms are trying to integrate large numbers of IoT devices in their systems. For this reason, there is already some research done for fault detection in IoT systems. IoTRepair [34] is a fault diagnosis system for IoT systems. Its diagnosis is facilitated by developer configuration files along with user preferences and works by monitoring the states of each sensor and how they correlate with the states of their neighbours. Power and Kotonya [35] provide an architecture with micro-services for fault diagnosis, through event handling and online machine learning, as a two-step approach. To provide a reasonable sensor value in case of faults, different imputation techniques are defined in the literature. Izonin et al. [36] developed a missing data recovery method by using Adaboost regression on transformed sensor data through Itô decomposition and compared the results with other algorithms like Support Vector Regression (SVR), Stochastic Gradient Descent (SGD) regressor, etc. Liu et al. [37] defined a procedure to deal with large patches of faulty data in uni-variate time-series data. Al-Milli and Almobaideen [38] proposed a recurrent Jordan neural network with weight optimisation through genetic algorithms. Most of the techniques that are used for the detection and recovery of faults are computationally expensive techniques that would evidently become a burden on the processing units with the increase of devices in IoT systems. In contrast to the aforementioned approaches for a search engine for the IoT that can be used in cross-domain scenarios, an objective approach to calculate the quality of received information is presented in this work.

### 2.5. Indexing of Discovered Resources

The large volumes of heterogeneous and dynamic IoT data sources that are available nowadays should be indexed in a distributed and scalable way in order to provide fast retrieval to user queries [39]. Depending on the attributes to be indexed, different techniques are required. For location attributes, the work in [40] proposed a framework that supports spatial indexing of the geographic values of data collected from sensing devices based on geohash (Z-order curve). Barnaghi et al. [41] combines the use of geohashing and the semantic annotation of sensor data for creating a spatio-temporal indexing. Before applying the k-means clustering algorithm to distribute data in the repository and allow data query, dimensionality reduction is performed to the geohash vectors by means of Singular Value Decomposition (SVD). An index structure is proposed in [42]. The process starts by clustering the resources based on their spatial characteristics and creating a tree structure in each cluster, where each branch represents a type of resource (e.g., humidity or CO${}_{2}$ sensors). The most notable works that are used for indexing time series are Symbolic Aggregate Approximation (SAX) and its variants (e.g., iSAX 2.0 [43] and adaptive iSAX [44]). A great deal of IoT data can be considered as a time-series, since by nature each observation will have a timestamp associated to it. These methods consider that the data follow a Gaussian distribution and use z normalisation processing, by which the magnitude of data vanishes. Since IoT data do not necessarily follow the Gaussian distribution and/or due to concept drift, the data distribution may change over time, SensorSAX [45] adapts the window size of the data according to its standard deviation in a online manner. Another work that is relevant in this sense is Blocks of Eigenvalues Algorithm for Time Series Segmentation (BEATS) [46], since it uses a non-normalized algorithm for constructing the segment representation of the time-series raw data. The mentioned methods, derived from SAX, are used to convert raw sensor data into symbolic representations and to infer higher level abstractions (for example, dark rooms or warm environments).

### 2.6. Ranking of Search Results

While the index cares for fast retrieval of search results, users and applications might still face the problem of sorting through a potentially large number of search results. Ranking mechanisms can help to sort and prioritise resources and services by selecting the most suitable one. In the Web domain, Google’s PageRank [47] is probably one of the most notable ranking algorithms. PageRank explores the links among Web pages to assign scores to documents, which are used in combination with text similarity metrics in the context of Web document search. In the IoT domain, on the other hand, the definition of similarity varies and resources can relate to each other based on a number of different features such as their type or their location. Not only the number of features for IoT resources can vary, but also the notion of similarity itself. Therefore, IoT ranking requires a multi-objective decision-making process in which the criteria to be considered are heavily dependent on the application and the domain. There exists work that already explores the multi-criteria nature of IoT domains for assigning ranking scores [39]. Guinard et al. [48] propose a ranking method for IoT resources which takes into account the resources’ type (e.g., temperature), their multi-dimensional attributes (e.g., location) and/or the Quality of Service (QoS) (e.g., latency), and applies different ranking strategies for multi-criteria evaluation with different criteria weights which are determined by the query (e.g., 40% for location, 40% for resource type and 20% for network latency). The work in [49] ranks sensor services based on two different QoS categories in Wireless Sensor Network (WSN), namely network-based (bandwidth, delay, latency, reliability and throughput) and sensor-based (accuracy, cost and trust). Other works incorporate user feedback/rating into their ranking mechanisms [50,51]. In IoTCrawler, we have devised a ranking method which can be tailored to the different applications.

## 3. Search Framework for IoT

In contrast to web search engines, a search engine for the IoT is used mainly by other machines or applications that need information to work properly. While a human user has the ability to assess the usability of a search result to his needs, a machine is not able to do so. It is expected that all search results returned satisfy the search query, as there is no objective way to decide between them. Therefore, an IoT search engine should rank the results beforehand, even without specifically stated requirements within the search query. For this, it should use all available information about the IoT device, such as long-term availability and reliability. Search results for a human can be presented in different ways. Not only text-based results, but also images, tables and videos are popular ways to transfer knowledge. A machine, in contrast, requires not only a fixed endpoint, but also predefined data formats. It needs to know beforehand how to interpret a received search result as well as the IoT data stream.

Like with any conventional search engine, looking for available resources at the time a search request was issued is not feasible. To provide search results in a timely manner, a data repository or database about the data sources needs to be built in advance. To further decrease the search time, the data within the database needs to be setup with appropriate indices. For example, as the location of a device is an important factor when searching the IoT device, providing indices related to the location can significantly improve the search. Before all of that, the search engine needs to be aware of IoT devices. This is probably the most challenging task since there exists a variety of different IoT devices and configuration possibilities. In addition, the IoT domain is more dynamic than the World Wide Web. While web servers usually remain online and stationary over a long period of time, the IoT devices may appear and disappear frequently. Thus, once an IoT device has been identified and integrated into the search engine’s database, it needs to be monitored for availability and stream quality. At the same time, the environmental context of the IoT device can change, which needs to be captured to provide additional search criteria.

For the IoTCrawler framework we adopted the search concept for IoT into the following two steps: (a) by presenting the Crawling and Processing Layer and (b) by presenting an incoming search request into the Search and Orchestration Layer. The parts labelled with a number (1–5) belong to the former layer and the ones labelled with an alphabetical character (A–D) belong to the later layer.

The **Crawling and Processing Layer** is the “online” part of the framework. It is constantly running and responsible for integrating new data sources into the framework. In this first step (1), data sources of different kinds are found and integrated in the MDR level. The federated MDR is the anchor point of the IoTCrawler framework (cf. Section 4.2), and holds metadata information for all data streams available in the framework. In step (2), the IoTCrawler information model is applied. IoTCrawler features an extensive Information Model based on the Next Generation Service Interface for Linked Data (NGSI-LD) standard and centred around the concept of IoTStreams [14]. The model provides the basis for the information stored in the MDRs and the integration of heterogeneous data sources. Both steps enable other parts of the framework to handle heterogeneous data sources. After the integration of new data sources, the SE comes into play (3) to further add new information to the data sources. The SE component enriches known data sources with new information extracted from the received data. The SE includes a quality analysis component that adds QoI (cf. Section 4.4.1) as well as a Pattern Extractor (PE) (cf. Section 4.4.2), which analyses data and provides higher level information.

In parallel, the enriched data (streams) are monitored (4) to enable the Fault Detection (FD) and Fault Recovery (FR) solutions of the framework. The Monitoring component ensures a constant user experience by detecting faulty streams and providing data recovery mechanisms (cf. Section 4.3). In addition, it features a virtual sensor creator to replace faulty data streams by an ML-based virtual copy. In the last step (5), within this layer, the search indices are created, allowing data sources to be found in the search process in a fast manner. The Indexing component is directly supporting the search of data streams by building indexes for the stream types and their attributes, such as locations (cf. Section 4.5).

The **Search and Orchestration Layer** contains components for handling search and subscription requests coming from IoT applications or individual users (A). The Orchestrator (B) is the main entry point for any user or application that wants to search for IoT devices (cf. Section 5.4). It organises the search process and orchestrates the needed data streams. The Orchestrator utilises the Search Enabler component (C), to resolve context-aware GraphQL requests to NGSI-LD requests and thus providing an easy-to-use interface hiding the complex NGSI-LD query mechanisms. For subscription requests coming from IoT applications, the Orchestrator can process the information gathered from the Search Enabler and is able to provide an endpoint to receive notifications about the stream properties, e.g., detected faults. NGSI-LD requests are redirected to the Ranking (D) component, which uses the built indices, given (user) constraints, and enriched information to rank the found data sources before they are sent back to the user or application.

**Figure 1:**
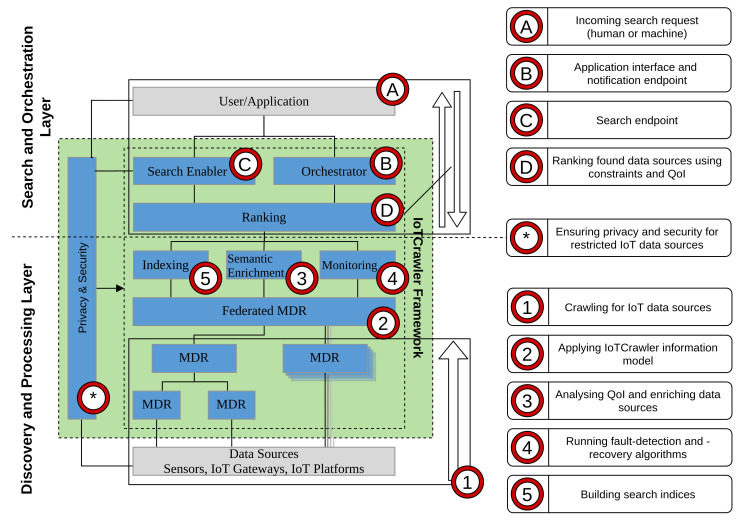
IoTCrawler addressing search in Internet of Things (IoT).

All steps, in both upper and lower layers, are constantly supported by IoTCrawler’s Privacy and Security components (cf. Section 5.1) to continuously ensure restricted access to IoT data sources for legitimate users (indicated with a * in Figure 1).

IoTCrawler enables users and applications to search for data sources, while addressing the challenges mentioned before. Due to the loose coupling of components via publish and subscribe APIs and the design of the single components, the framework is designed to reach high scalability (R-1).

## 4. Enablers for Discovery and Processing Layer

This section addresses enablers for the discovery in the IoTCrawler framework, introduced in Section 3. A detailed description for each enabler is provided and complemented by an evaluation on the enabler’s performance.

### 4.1. Information Model

**Figure 2:**
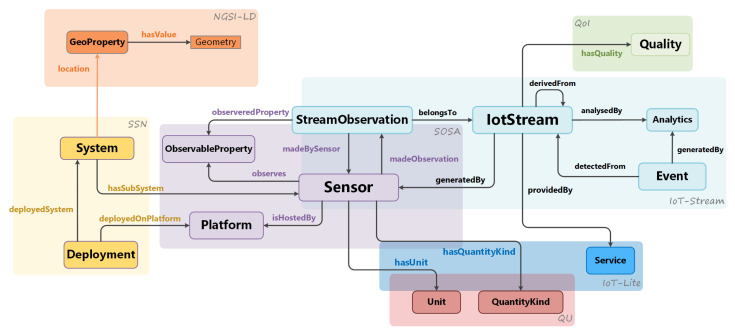
IoTCrawler information model.

The IoTCrawler information model is built upon standards. It follows the NGSI-LD standard and combines it with well-known ontologies, to reflect IoT use cases in the context of IoTCrawler and to address the requirement of semantics (R-2) to provide machine readable results. The choice of NGSI-LD is justified by several factors: being based on an standard makes it easier to inter-operate with, to integrate with other technologies and to maintain and evolve. Added to that, NGSI-LD supports semantics from the ground-up, which is one of the core strengths of IoTCrawler and an enabler for some of the functionalities that it provides. NGSI-LD provides not only the incorporation of semantic information to the data, but also a core information model and a common API to interact with that information (commonly called context). It was chosen as the main anchor point for the interactions between the components in IoTCrawler, greatly reducing and simplifying the number of different APIs to implement and keep track of, as well as data formats and models. This “common language” not only serves an internal purpose to simplify and optimise, but also makes IoTCrawler components easier to be integrated outside of IoTCrawler itself, and has already allowed to integrate existing components (like the MDR) seamlessly into IoTCrawler. The model has been designed to capture a domain that focuses primarily on sensors and stream observations. To achieve this and following best practises [52], concepts were reused from the SOSA ontology [11]. To enable search based on phenomena, the ObservableProperty is also reused. The Platform class is used to capture where the Sensor is hosted on. In addition to SOSA, the SSN ontology is used to capture what Systems sensors belong to and where they are deployed. Although SOSA captures concepts for sensors and observations, the concept of streams is missing, which is a fundamental aspect for IoTCrawler as it involves stream processing. For this, the IoT-Stream ontology provides the concept by defining an IotStream [14]. The IotStream class represents the data stream that is generatedBy the sensor as an entity. It also extends the SOSA ontology by defining a subclass of the Observation class, StreamObservation. This has been done to extend the temporal properties of an Observation to include windows as well as time points. For accessing the Service exposing the IotStream, the Service class from the IoT-lite ontology is used, and this enables direct invocation of the data source. The IoT-Stream ontology also provides concepts for Analytics and Events, which represent aspects of the semantic enrichment process. Moreover, with regard to the semantic enrichment process, IoT-Streams link to external concepts that capture QoI information about the streams. The QoI ontology provides this, which captures aspects of quality such as Age, Artificiality, Completeness, Concordance, Frequency and Plausibility [53]. An important aspect to any entity is location. Here, the NGSI-LD meta-model which defines a GeoProperty is used. The main classes and relationships of the IoTCrawler model are illustrated in Figure 2.

### 4.2. Federation of Metadata Repositories

A key enabler in the IoTCrawler framework is the federation of multiple MDRs. The MDRs stores all available metadata information gathered by the discovery process. Considering the requirements from Section 1, the MDR has not only to support the IoT search as a whole, but also to address the requirements for scalability (R-1) and semantics to allow machines and applications to use available IoT data sources (R-2).

In addition to other technologies, e.g., triple stores or relational databases, IoTCrawler has chosen to use the NGSI-LD standard, which not only defines a data model for context information forming the basis for IoTCrawler’s data model (see Section 4.1), but also defines an API, which will be used by consumers and providers alike, to access information. Among the API functionalities offered by the MDR are: the direct query and publish/subscribe mechanisms, which allow context consumers to receive notifications whenever new information is made available in the system. This publish/subscribe mechanism is extensively used in IoTCrawler for communication and synchronisation between different components, which will subscribe to context information relevant for their purpose, and will publish the processed information to make it available to other components.

**Figure 3:**
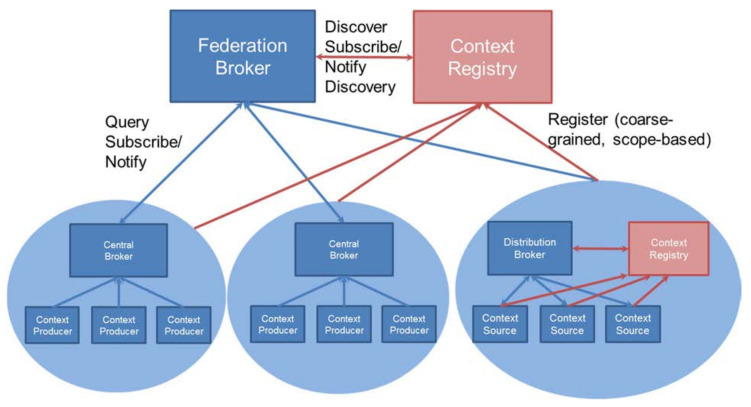
Federated broker architecture [54].

NGSI-LD brokers can be interconnected in different ways to achieve scalability. The most best performing deployment configuration of NGSI-LD brokers, which is used in the IoTCrawler framework, is the federated one as shown in Figure 3. It consists of a federation of brokers, in which all information of the different federated brokers is accessible automatically through the federation broker. This last broker acts as the central point of IoTCrawler’s architecture and is the key in making IoTCrawler horizontally scalable and well performing. This allows all other components in IoTCrawler to use the MDR in a scalable and standardised way and, being based on the NGSI-LD standard, not only makes the MDR inter-operable and compliant to standards, but also allows for the use of different already existing implementations.

For our current deployment, we have used Scorpio (https://github.com/ScorpioBroker/ScorpioBroker accessed on 24 February 2021) because it is the only implementation which considers a federated scenario. Nevertheless, in the frame of this paper we have focused our metrics on a single instance of this broker, obtaining both latency and scalability metrics. To do so, we have deployed a virtual machine with the following features: 8 CPU cores and 28 GB of RAM inside a Google cloud. Latency has been evaluated over the different operations provided by the MDR, specifically: entity management, publication/subscription and context provisioning.

Measurements show that the most time consuming operation is the process of getting entities specified by their ID, which takes around 800 ms. This operation should not be so cumbersome and we think that the low performance associated with this task could be due to the maturity of this software. Apart from this operation, the rest of the operations take from 17 to 37 ms to perform, which is a more affordable processing time. Regarding subscription management, the creation of subscriptions is a heavier task taking up to 270 ms, whereas the other operations take only about 17 ms. Finally, context provisioning tasks, which comprise the registration of the information coming from context providers pointing at the end-point services provided by them, take more time compared to the previous tasks. Nevertheless, the registration and deletion of context providers are operations which are usually executed once per context provider. By contrast, the operations to obtain context providers take about 100 ms.

**Figure 4:**
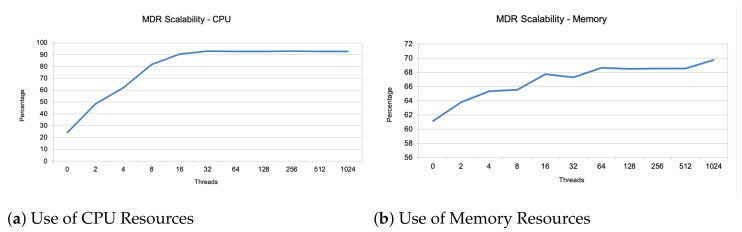
Metadata Repository (MDR) scalability assessment.

Finally, regarding the scalability metric, we have focused on the CPU and memory resources consumed by the instance of the NGSI-LD broker according to a specific range of simultaneous connections (2, 4, 8, 16, 32, 64, 128, 256, 512 and finally 1024). In addition, we have repeated this process four times. The results of these tests are presented in Figure 4, depicting that the CPU resources’ consumption follows a logarithmic curve where the steepness of the slope is lowered from 8 simultaneous communications on. On the other hand, we can see that the increase in simultaneous communication does not impair the memory resources notably.

### 4.3. Monitoring

IoTCrawler allows aforementioned participants to connect their sensors to the system, to make them available for a broader audience. As sensors are often deployed in environments where their operation cannot be controlled or even guaranteed, and given that many of them are battery powered and have a limited life span, it is to be expected that their reliability might fluctuate over time. It is therefore important to observe the performance of the sensors. For this, IoTCrawler has developed the Monitoring component, which is responsible for observing the incoming data streams of different sensors, detecting possible faults in the data, and, if possible, providing counter measures to mitigate them. The proposed monitoring concept with its different subcomponents provides an extensive set of features for addressing issues of dynamics in IoT environments.

#### 4.3.1. Fault Detection and Fault Recovery

The Fault Detection (FD) component monitors the data streams that are available to the IoTCrawler framework and follows a two-layered approach. In the first layer, the component categorises faults as definite faults (due to hardware issues) or as anomalies, which could occur because of brief environmental factors, an unexpected behaviour detected through learned patterns. These anomalies can be categorised as faults, if they persist for a longer period of time. To cater to the needs of most of the sensor streams, the FD component uses different algorithms, e.g., the Prophet algorithm [55] for time series analysis and stochastic algorithms which determines the likelihood for a value to occur based on the previous observations of the sensor. The FD component subscribes to new data streams that become available through the MDR. Through the metadata, the FD determines which approach should be used. This is differentiated based on how much information is provided in metadata. The MDR is then notified in case of faults and trigger the recovery mechanism. To deal with faulty sensors, IoTCrawler has developed a two-stage counter measure. The Fault Recovery (FR) mechanism is a first response to handle missing sensor observations by imputing artificially generated sensor readings. The goal here is to have a quick solution to provide uninterrupted data streams for the applications using them. For long-lasting faults, the FD can issue the deployment of a virtual sensor to replace the broken one.

In the case of multiple sensors, we employ a knowledge-based Bayesian Maximum Estimation (BME) for imputing an identified faulty value [56]. BME is a mapping method for spatiotemporal estimation that allows various knowledge bases to be incorporated in a logical manner—definite rules for prior information, hard (high precision) and soft (low precision) data into modelling [57]. More details about this algorithm can be checked in [56].

**Figure 5:**
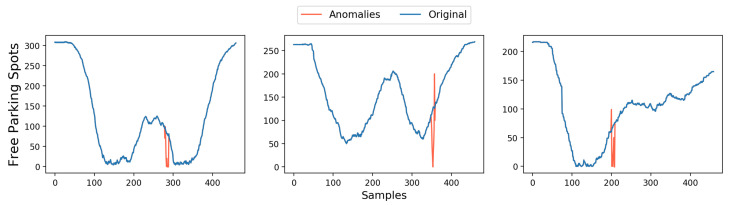
Data with injected anomalies at different instances.

To evaluate the working of FD and FR, an instance of FD is presented in the example below. Sensors deployed in three different parking areas in the city of Murcia are integrated into the IoTCrawler framework. These sensors record the information about the number of free parking spots in their respective parking lots with an update interval of 2 min. A model was trained on the data of several days from the parking areas to learn the normal behaviour. As an instance of the results, Figure 5 shows the original data for one day from each sensor, each along with one injected anomaly.

The algorithm detected two anomalous patches in each instance. The first detected patch consists of the initial samples where the values do not change and the second anomalous patch is the actual anomalies. The first fault in each instance, caused by the sensor, is considered to be in stuck-at fault condition as this behaviour was not observed in the training set. The stuck-at condition is fulfilled when a sensor repeats an observation more times than was observed in the training set.

**Figure 6:**
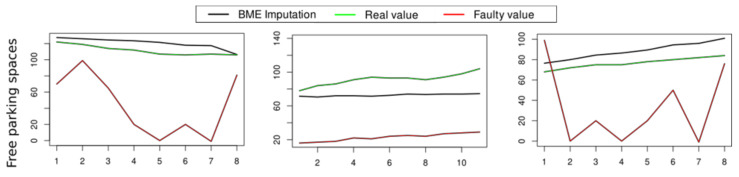
Comparison of actual and recovery values at the anomalous patch.

For the stuck-at fault, an estimated value cannot be interpolated by the neighbouring sensors, as all of the sensors show the same faulty behaviour at the same instance. The second anomalous patch in each sensor occurs when the data from another sensor is normal at those time instances. A recovery value is then generated using data from sensors with normal behaviour and BME as the interpolation technique (explained above). Results can be seen in Figure 6.

#### 4.3.2. Virtual Sensor

To replace a faulty sensor in the longer term, IoTCrawler provides the virtual sensor component. A virtual sensor is capable of providing artificial sensor observations for a longer time as it is trained on larger data sets with different algorithms. As a result of the FR mechanisms as a first response, virtual sensors are allowed to train for a longer period of time, hence allowing the algorithms to learn more patterns which also make them capable of learning data drift. The concept of virtual sensors is that it takes historical data from a broken sensor and its correlating sensors and use the relationship to predict the values in place of the broken sensor. For instance, in the case of a broken temperature sensor, a virtual sensor can be trained to project the temperature at the failed sensor’s location using nearby temperature sensors as predictors. To achieve this, the component searches for neighbouring sensors that can be used as predictors in the ML model. The correlation between the broken sensor and each candidate is calculated to train only with the most promising data sets. Via a grid search approach, the most promising ML model is selected.

To test the component, different scenarios are considered and the results are documented in [58]. The viability of virtual sensors has been shown in different environments by considering neighbouring sensors with the same and different sensor types than the faulty one. Models selected through grid searching along with models created through ensembling were used to make the predictions, both of which showed promising solutions. The results show that a fully autonomous deployment of virtual sensors is possible, although it should be mentioned that their effectiveness highly depends on the availability of correlating surrounding sensors.

### 4.4. Semantic Enrichment

The IoTCrawler framework is capable of adding new meta-information to known data and data sources. For this purpose, the Semantic Enrichment (SE) is being used. Currently, the component contains two parts, but can be extended further: the QoI Analyser and the Pattern Extractor, where the first one is responsible to add information about QoI to a data stream and the second one to extract patterns and therefore learn additional information from a stream.

#### 4.4.1. QoI Analyser

The QoI Analyser is responsible to annotate data streams within the MDR with additional QoI metadata. By combining metadata and predefined QoI metrics, it is possible to rate incoming data from data streams and therefore to annotate these streams with QoI. This additional information about quality enables other components of the framework to provide (better) results, especially the Monitoring (cf. Section 4.3) and the Ranking (cf. Section 5.2) components.

An important step is the definition of QoI metrics that are available within the IoTCrawler framework. Currently, the QoI Analyser supports five QoI metrics that have been defined: Completeness, Age, Frequency, Plausibility, Concordance and Artificiality. For details and calculation of the QoI metrics, we refer to [59,60,61]. To integrate the results of the QoI calculation an ontology has been created and integrated into the information model as shown in Section 4.1.

**Figure 7:**
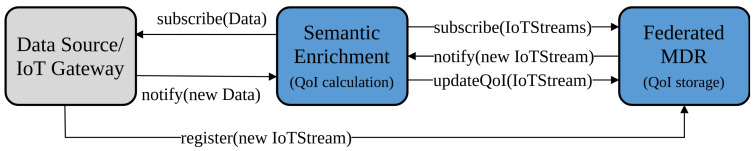
Semantic Enrichment (SE)–MDR communication.

A main anchor point for the integration is the publish/subscribe interface provided by the MDR. Figure 7 provides an overview of the interactions of the QoI Analyser and the IoTCrawler framework. When a data source is registered or updated at the MDR, the registration contains additional metadata, e.g., a detailed description of the data sources properties and its characteristics. This allows to adopt the QoI calculation to changes in the metadata or to connect to a new data endpoint description to access data. Finally, the QoI Analyser calculates the QoI for each known data source and adds the results to the metadata. This allows other IoTCrawler components as well as third-party users to access the additional information.

**Figure 8:**
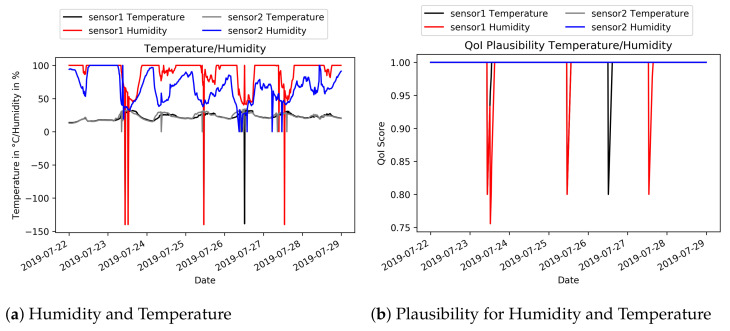
Aarhus CityProbe sensor’s data and Plausibility.

For the following experiment, data from the city of Aarhus, Denmark are used. The data set named “CityProbe” is a real-time data source, which consists of 24 sensors that are mounted on light poles. The devices are solar powered and provide different sensor values, e.g., humidity, temperature, rain or CO. These data are analysed and it is shown how the QoI Analyser detects increasing or decreasing quality of the incoming data. For the experiment, the metadata annotation has been set to the following values: The range for the measured temperature has been set to −30 ${}^{\circ }$C to 40 ${}^{\circ }$C, which depicts a common temperature range for a northern country, whereas the humidity ranges from 0% to 100%. These ranges are used for the calculation of the Plausibility metric by checking if the observations remain in the defined ranges. Figure 8 shows an analysis of two sensor devices for temperature and humidity data for a time span of one week. The first graph depicts the measured values, whereas the second one shows the calculated Plausibility values. Figure 8a shows some suspicious temperature and/or humidity peaks in the minus area. From a human point of view, they can assumed to be wrong. In addition, the Plausibility metric decreases as it can be seen in Figure 8b. This example shows a use case of a decreasing QoI metric. A possible subscriber of the QoI, e.g., the Monitoring, can now react to the dropping information quality. In case of the Monitoring, it is now possible to initialise a more complex FD or FR algorithm or to create a new virtual sensor instance.

With the QoI Analyser, it is possible to identify data streams with decreasing quality. As an example, the Frequency metric is able to detect if a data stream does not provide data in the annotated time interval. Of course, it is not possible to directly detect the reason for a decreasing Frequency as IoTCrawler has no access to the sensor devices, but it provides the results of the QoI calculation to other components, which can then react to a changing QoI, e.g., by selecting an alternative data source. With that, the QoI Analyser enhances the Reliability in IoT environments. The QoI annotations also give objective criteria to choose between data streams, especially when the search is performed by a non-human system (Requirement R-2).

#### 4.4.2. Pattern Extractor

To allow context-based search (Requirement R-2), the Pattern Extractor (PE) module enables the generation of higher-level context The context itself would be defined by the domain(s) of interest of the deployment, e.g., traffic congestion levels or personal health activity monitoring. The PE relies on a pre-training process in which it creates a set of clusters, each corresponding to a state or event. The PE analyses annotated IoT data streams that are pushed to the Metadata Repository to detect Events, by employing a data analysis technique. A subscription to the MDR is made for StreamObservations that have a certain property, and can also include spatial and temporal filters. iot-stream:StreamObservations of iot-stream:IotStreams that meet the requirements are then pushed as notifications the PE component. The PE temporarily stores a certain number of observations that correspond to the time window pre-defined by the deployer. The output of the analysis is a textual label that interprets the pattern of data. The label is then encapsulated in an iot-stream:Event instance, along with the start and end times of the window in question, and published to the MDR.

The algorithm for pattern extraction is based on aggregating observations from a time window for pattern representation. Observations are grouped in time windows of predetermined size. On each window, Lagrangian Pattern Representation (LPR) [62,63] is applied to determine the patterns. Patterns are then clustered and grouped using Gaussian Mixture Models (GMM). The number of clusters depends on the number of expected events for a specific scenario. A label representing the pattern is given to each cluster. Label nomenclature is defined by the topical domain ontology for the specific use case.

In the PE component, there are two models that represent patterns [63]. K-means clustering was used for the first approach of representing patterns and our model applied to some data sets from UCR Time-series Classification Archive [64], which is known as a benchmark data set for clustering and classification methods. The data sets Arrowhead, Lightning7, Coffee, Ford A and Proximal Phalanx Outline Age Group from the time-series archive were used. The Arrowhead data set contains shapes of projectile points in time series. Lightning7 has data of time-domain electromagnetic from lightnings. The Coffee data set contains data from measurements of infrared radiation interaction with coffee beans, which is used to verify the coffee species. Ford A has measurements of car engine noise and Proximal Phalanx Outline Age Group has observations from radiography images from hands and bones. Silhouette coefficient was used to evaluate the model. Silhouette is a measure of how separated the constructed clusters are from each other. To evaluate the clustering technique in the real-world scenario, we need to use a measurement to evaluate the separation of the clusters as we do not have the true classes. The results were compared by using K-means on raw data without Lagrangian representation. Table 1 proves that our method improves the clustering results of these data sets.

**Figure 9:**
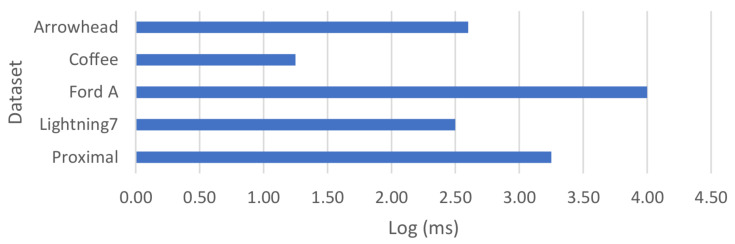
Processing time of the clustering algorithm for different data sets.

The measurements for the above data sets were conducted using a machine with a 4.00 GHz 4-core CPU and 32 GB of RAM. In the case of the time series in the Ford A data set, the averaging processing time for applying LPR on it was between 400–500 milliseconds. Figure 9 shows the relative comparison of the clustering algorithm processing time applied to each data set.

For the evaluation of Principal Component Analysis (PCA)-Lagrangian representation, the method was applied to both synthetic and real-world data. GMM was then used for clustering. We generated a synthetic data set using a multivariate Gaussian distribution and generated a time series including 2400 samples with four dimensions with three different Gaussian distributions which have the same covariance matrix and different mean vectors. Each distribution had 800 samples. In addition, another data set was generated by adding white noise with Signal-to-Noise Ratio (SNR) of 0.01. The results of the Silhouette coefficient are 0.87 for data w/o noise and 0.47 for data with noise.

For a real-world scenario, we used air quality data from Aarhus’ open data. We used air quality data from a period of two months with a sampling frequency of every five minutes. The data have two dimensions; Nitrogen-dioxide (NO${}_{2}$) and Particulate Matter (PM). There are three different clusters: low risk, medium risk and high risk. We evaluated the results using Silhouette coefficient and compared the results. The results are shown in Table 2.

The proposed algorithms for pattern extraction allow to extract high level events directly from the IoTCrawler framework (R-2). They also reduce the need for external applications to subscribe to raw data and decrease the amount of transferred data, improving scalability (R-1).

### 4.5. Indexing

**Figure 10:**
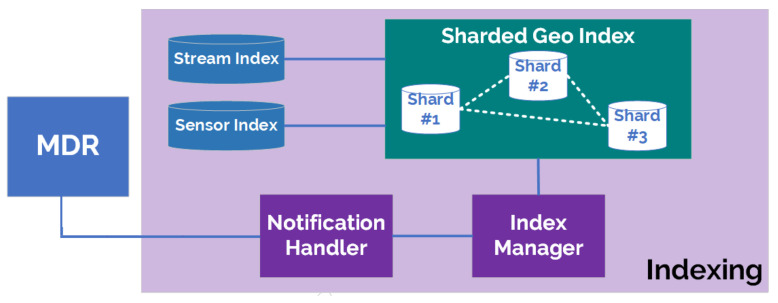
Indexing sensors and IoTStreams.

Indexing provides a means for clients to search for IoT entities efficiently. It focuses on IoT streams and sensors, where queries can be based on sensor type and absolute or relative location. To initiate the process of indexing, a platform manager needs to register a MDR with the Indexing component. In turn, this will trigger the subscription to sensors and streams at the registered MDR. As the metadata descriptions are updated at the MDR, the Indexing component will be notified and then index the sensors and streams based on location. For scalability, the Indexing component can be configured so that the persistence it relies on (MongoDB) can be shared (see Figure 10).

Indexing exposes a query interface which complies with the NGSI-LD specification. Upon querying by a client, entities that relate specifically to sensors, IoT streams, location points or QoI will be responded to directly. Else the query is the forwarded to the MDR for complete query resolution. The approach enhances the query resolution performance significantly, as co-located entities and common types are indexed and grouped, allowing reduced latency in query processing.

The indexing technique applied is based on a geospatial approach defined by Janeiko et al. [65]. The index is a tripartite whereby two of the indices link iot-stream:IoTStream and sosa:Sensor entities to a geo-partition key. The other index contains the actual data and is also geo-partitioned. The partition key is determined by intersecting the location of the sensor represented as GeoJSON objects with predefined GeoJSON polygons representing geographical regions. The index contains the entities in the form of a graph, whereby linked entities are stored as a single entry. Here, the IoTStream entity is the root entity with all other indexed entities are nested within it, hence any query for any entity must be linked to an IoTStream entity. The structure defined allows to construct compound indices, which accelerates nested queries. By providing its indices for search, the Indexing component addresses scalability R-1.

The Indexing component is responsible for creating and updating the metadata indices to allow fast search and retrieval of the metadata stored in the MDR, using geospatial indexing. The initial approach for geospatial indexing IoT Streams and Sensors was to use geohash, whereby the location is represented by a string of characters with a predefined length reflecting the granularity of the bounding box the entity will be associated with. A new approach has been taken to maintain the exact location of the entity by using a Quad Search Tree. The main KPI that is applicable is the latency and retrieval time. The Indexer partitions the notifications from MDR broker notifications for stream or sensor data location by country. Latency and retrieval time can be measured based on: a data set’s size or number of entities, i.e., streams and sensors, a number of countries or a number of concurrent requests.

Therefore, the approach to evaluation will be applied to a data set with different sizes, multiple countries. Data sets were randomly generated which covered entities located within 6 countries. In terms of hardware, the experiment was conducted on a computer with an Intel CORE i7 CPU of 6 cores, 1.9 GHz and 32 GB RAM. Concurrency tests were performed using the Apache Bench tool. Two sets of tests were performed. Each set had a number of entities stored in the indexer. For each set, two sets of concurrency tests were performed: one with 100 requests (the graphs show the total time for all requests) with a concurrency of 10; and 10,000 requests with a concurrency of 1000. Regarding the query response time, two factors are measured, the total time for the response, the wait time and the time the indexer receives the requests and responds, irrespective of the connection time.

**Figure 11:**
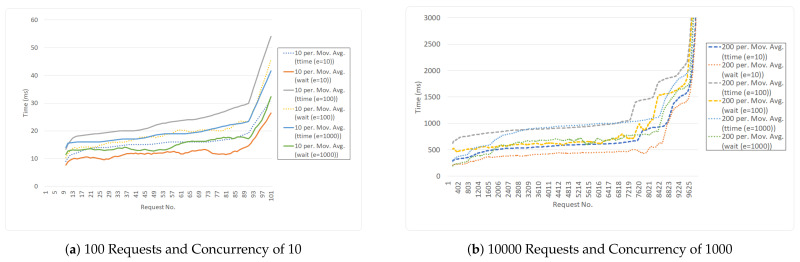
Indexing response times.

Between the 3 sets of tests, the wait- and total response times show a gradual increase with respect to the number of stored entities. What is also noted is that for the last set of concurrency requests, a significant change in delay is observed, especially in the case of requests with a concurrency of 1000. Figure 11a,b show the response times for requests with increasing concurrency. The plots have been smoothed out with a moving average of 10 and 20, respectively.

## 5. Enablers for Search and Orchestration Layer

This section addresses the enablers for search in the IoTCrawler framework. Based on the description in Section 3, all enablers will be shown in detail, including experimental results and evaluations.

### 5.1. Privacy and Security

The IoTCrawler framework places security and privacy as a traversal pillar interacting with the different layers of its architecture (cf. Figure 1). This pillar comprises: Identity Management (idM), access control management, for both intra-domain and inter-domain and finally privacy from a data point of view. Starting with idM, this component is responsible for handling the different identities that are registered in the IoTCrawler framework. An identity, which can be a user, device or service comprises different attributes such as: name, email, role and organisation, to name a few. They are quite important for the definition of access control and privacy encryption policies as we will see below. Another important function carried out by the idM is that of authentication. Any entity registered in the system must perform the login operation due to the exposed API. In our case, we have selected the FIWARE KeyRock GE (https://fiware-idm.readthedocs.io/en/latest/ accessed on 24 February 2021), which exposes an OAuth2 API.

To deal with this heterogeneous landscape, we have designed a comprehensive approach where we are combining a distributed authorisation solution called Distributed Capability-Based Access Control (DCapBAC) with Distributed Ledger Technology (DLT), specifically Hyper Ledger Fabric (https://www.hyperledger.org/use/fabric accessed on 24 February 2021) and the use of smart contracts. DCapBAC decouples traditional authorisation solutions, such as XACML framework, in two different phases: authorisation request and access. For that, a new component, called Capability Manager (CM), is introduced. It is the end-point for the authorisation requests and it also issues an authorisation token called Capability Token (CT) after validating the authorisation request by communicating with the XACML framework. Regarding the access phase, the XACML Policy Enforcement Point (PEP) is moved as a Proxy located close to the server where resources are stored. In this case, CT acts as a proof of possession which allows the PEP Proxy to validate it easily without querying any other third party. This CT contains all details regarding the resources to be accessed, the access mode among others.

**Figure 12:**
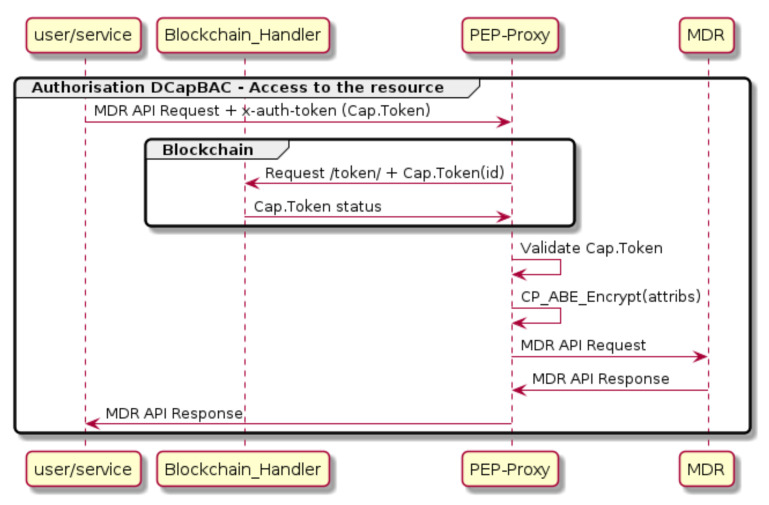
Policy Enforcement Point (PEP) Proxy interaction diagram.

DLT provides numerous advantages in term of resilience, and traceability because of its consensus approach where all nodes of the network must agree on global policies. For this reason, in IoTCrawler, an additional step is taken as showcased in Figure 12, by introducing the Blockchain as an added element in the security process; by storing policies in the Blockchain, as well as CTs that can later be revoked; and thus need to be checked by the PEP Proxy in the Blockchain for validity.

Access control components are integrated into the Blockchain to enhance security and scalability. By leveraging Blockchain, several issues of current access control systems can be overcome.

- Untrustworthy entities: First, Policy Administration Point (PAP) might be subject to an attack and perform malicious actions such as updating a policy against the resource owner’s will. Having a Blockchain helps avoid misbehaviour of PAP. The access control policy’s integrity is checked by registering and checking its meta-data, such as the hash value managed by the Blockchain network. Second, policy evaluation done by Policy Decision Point (PDP), which could be manipulated by an untrusted PAP. The Blockchain ensures this misbehaviour to be detectable.
- Auditability: The verifiable property of Blockchain allows detecting if an access control service falsely denied access to a subject that the policy would grant or if the access control service granted a permission while the policy was not satisfied.
- Revocability: The attribute-based access control model that we have in this framework assumes, once a subject has granted an access permission, that the subject will receive an access token. It is challenging to revoke the token once it has been misused or stolen. Blockchain resolves this issue by executing a token smart contract to invalidate the vulnerable token.
- Fault tolerance: Access control components are distributed among peers over the Blockchain network. Such components are PAP, PDP and CM, among others. By having functions executed as smart contracts and invoked by a peer of the network, it avoids becoming a single point of failure as it would be the case with traditional PAP, PDP or CM.
- Integrity: New changes may cause disruption of such services and therefore they should be done cautiously. No single individual can introduce changes. This property is essential in the network where the participants often do not trust each other.

To address the scalability requirements R-1, we carefully design the security components so that only critical parts are executed on-chain and other parts can be done off-chain. Policy and capability managing operations are on-chain with policy enforcement and identity management can be done off-chain or access to another service. In addition, we carefully select the consensus algorithm, which is one of the core parts of the Blockchain, so that it provides efficient throughput and latency performance. As a result, security and privacy enablers provide by-design secure access to IoT data thanks to the DCapBAC access control model in privacy-preserving using attribute-based encryption. DCapBAC is coupled with Blockchain to provide distributed trust among untrusted domains by agreeing on common policies and ensuring policies’ integrity. In addition, Blockchain offers transparency, auditability and fault tolerance to access control. Our chosen Blockchain deployment with sufficient consensus algorithm ensures low overhead, in another word, high scalability.

For the evaluation of these components we have measured the latency associated to each of the operations that these components perform to grant authentication and authorisation, as well as the performance metrics linked to the CPU and memory consumption of these operations by increasing the number of simultaneous requests up to 2048 connections. We ran the benchmark experiment on a server with Intel Xeon E-2146G CPU, 32GB RAM, in a local network environment.

#### 5.1.1. Identity Management and Authentication Evaluation

**Figure 13:**
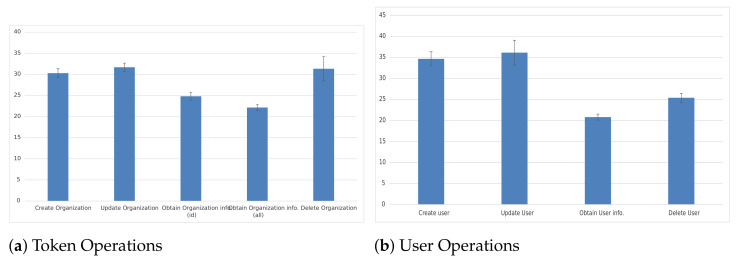
Delay of Identity Management Operations (units in milliseconds).

Starting with Keyrock, we have evaluated the latency on two different sets of operations, the first one is related to the generation, information retrieval and deletion of the Identity Management Token, while the second set is focused on the user point of view, providing information about common operations related to user management. For the evaluation of this metric, we have launched 100 executions of these operations to provide the average latency value and 95% confidence intervals as presented in the following graphs and tables. As we can see in Figure 13a, the delay obtained for the difference is really low, reaching up to 30 ms for the generation of the idM token. This operation, compared to the others, is the heaviest one because it comprises the different mathematical operations required to generate the token. The most common authentication operations usually triggered via web interface or REST API are shown in Figure 13b. In light of these results, we can also state that user operations last about 30 ms, which is reasonable in terms of latency.

**Figure 14:**
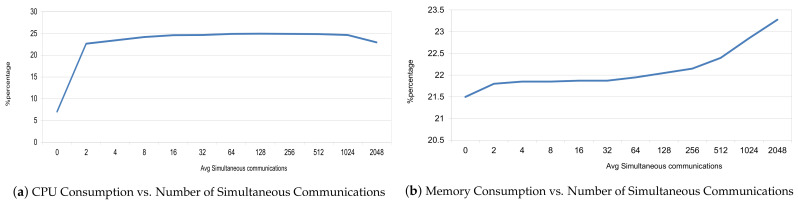
Identity Management Resources consumption.

Regarding the scalability aspect, we have assessed the performance of the idM in terms of CPU and memory consumption resources. The objective was to provide a trend as the number of requests increases, so that we can provide an estimation for a higher number of simultaneous requests. Therefore, to achieve this goal we have launched different number of simultaneous connections: 2, 4, 8, 16, 32, 64, 128, 256, 512, 1024 and 2048. Additionally, we have repeated the experiments 4 times. More specifically, we have employed a query for authenticating a user. According to Figure 14a,b, we can state that the CPU resources managed by the idM remain stable as the simultaneous requests increase. Regarding the memory resources, we can see that up to 256 simultaneous connections, the increase is less than 1.5%. From that number on, the memory resources increase again about 1.5%. Therefore, we can state that it is able to handle a large number of communications.

#### 5.1.2. Authorisation Evaluation

Authorisation addresses two different scenarios, intra-domain and inter-domain scenarios. Regarding the former, DCapBAC has been implemented. From this point of view, we have assessed different metrics with the objective of measuring the time to grant access to a user to a specific resource, and also to measure the performance in terms of simultaneous connections.

Consider thePDP request, which is the XACML validation process that is performed by the PDP after receiving the authorisation request coming from the CM. This takes around 200 ms. The CT generation considers the previous task, and includes also the processing time required by the CM to issue a CT, which takes about 1.6 s. Finally, the overall authorisation process from the point of view of the clients from the moment they issue an authorisation request, to the moment they receive the authorisation answer together with the CT was measured with around 1.65 s. Since the token includes a validity period to the resources to be accessed, issuing a CT is not required for every access.

**Figure 15:**
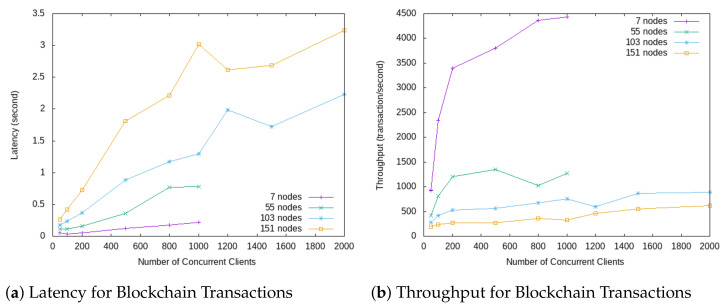
Latency and throughput performance for Blockchain transactions.

The DLT operates using Hyperledger Fabric framework with the Kafka consensus algorithm. The most essential and critical factor that affects the overall performance of a Blockchain network is the ordering service. Ordering service is a part of the consensus protocol. It generates a unique ordered sequence of transactions in a block and the block is delivered to nodes. We measured the Blockchain latency and throughput as the primary performance metrics for Blockchain, with various parameter settings of network size (number of ordering nodes) and block size (blocks committed to the Blockchain of each transaction). Ordering latency is the time a transaction needs to wait for the ordering service until its order in a block is assigned. Ordering throughput is the capacity the service can handle a certain number of transactions per second. Figure 15a,b show benchmark results of our Blockchain network. With small size network (7 ordering nodes), latency is very low (less than 0.5 s for 1000 concurrent clients). When the size of the network increases, the latency also increases (at size of 151 nodes, the latency at 1000 concurrent clients is 3 s). The same pattern for throughput performance. Throughput drops when network size grows (at size of 151 nodes, throughput at 1000 concurrent clients goes below 500 transaction/second). These benchmark results show the tradeoff between latency/throughput performance and network resilience against faulty requests. When the network size is larger it is more resilient to tolerate faulty nodes, however, it bears higher latency and lower throughput.

We have excluded the cost for the execution of transactions in the benchmark. Instead, we vary the block sizes which simulate various application scenarios in practice. Figure 15a,b show results at a typical block size of 100 bytes. For other block sizes, the patterns of latency and throughput hold the same. The overall performance of Blockchain network needs to consider also the transaction execution time, smart contract invocation time and transaction validation cost, which are application dependent.

### 5.2. Ranking

The Ranking component implements ranking mechanisms for IoT resources. Its purpose is to aid users and applications to not only find a set of resources relevant to their needs, but also to select the best or most appropriate one(s) from that set. There are multiple criteria for ranking IoT resources such as data type, proximity, latency and availability. Therefore, IoTCrawler’s Ranking component supports application-dependent, multi-criteria ranking. Within the IoTCrawler framework, the Ranking component is available to the Search Enabler component to facilitate entity discovery. The Ranking component relies on an NGSI-LD compliant endpoint as a backend, which is often times the Indexing component but could also be any NGSI-LD broker. Upon receiving a query request and its ranking criteria, the Ranking component initially forwards the query to the underlying index or broker to get the set of IoTStreams entities matching the query. A ranking function then computes, for each result, a ranking score, according to the ranking criteria. The score is then attached to each IoTStream result as an additional property. The ranking criteria specifies the relevance of different properties to the application. The current ranking function computes a weighted average of the QoI values of a IoTStream entity, where the weight values are specified in the ranking criteria, but it can be easily adapted to other ranking criteria. In this way, the ranking is addressing the requirement for search R-3 by successfully ordering the search results.

The Ranking component offers an extended NGSI-LD interface, where ranking criteria can be specified in addition to the query. To avoid any influence of indexing strategies implemented by the Indexing component and be able to focus on the performance of the Ranking component itself, we have evaluated the Ranking component in a simplified architecture consisting only of the Ranking component and an NGSI-LD broker. Although the Ranking component supports horizontal scaling (adding more instances behind a load balancer for better scalability) due to its stateless implementation, in this evaluation we have only tested on a single instance. To assess the scalability of the component, multiple queries have been sent, both directly to the broker and to the ranking + broker combination. For the ranking + broker combination, we used a single ranking weight as the ranking criterium, that means that results were sorted based on the value of a single property. We have varied the number of concurrent query requests and measured the latency in retrieving the results. Each request returned 1000 entities, where the entities’ size was approximately 7 kB.

**Figure 16:**
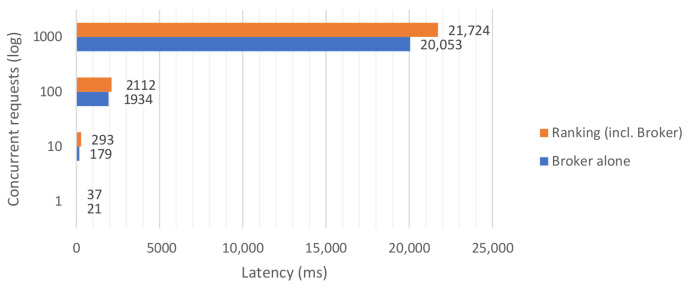
Ranking latency.

The results shown in Figure 16 indicate that the Ranking component introduces a small latency in retrieving the results, but it can nevertheless scale with the volume of query requests.

### 5.3. Search Enabler

The Search Enabler component is responsible for providing functionally rich query language and the search interface for seeking over metadata of discovered sensors and streams. Using GraphQL technology, the IoTCrawler search component offers end-to-end functionality for performing complex queries, allowing users to access data coming from distributed large-scale IoT deployments. Any complex GraphQL query is decomposed and resolved via a corresponding number of atomic NGSI-LD queries, as it is prescribed by the NGSI-LD standard. The schema-based approach of GraphQL allows to describe key entities (see IoTStream Ontology [14]) and the relationships between them. A compiled schema becomes a basis for query parser/validator engine and for a GUI, where users can design their queries. To comply with the linked data approach, all types and their properties in the schema are annotated with type URIs according to the IoTCrawler data model. Annotations describe hierarchical relations (equal to the subclassOf) between types, which are considered during query resolution process. This allows to be fully compliant with ontologies used for data modelling. For example, to describe a set of sensors hosted by a platform, a correct definition in terms of SOSA ontology would be: “system hosted by a platform”, which means that sensors, actuators and others subtypes belong to the more generic type used in this statement. Use of types and subtypes and considering their relations during query resolution process is an exclusive feature of the Search Enabler component developed for the IoTCrawler platform. Another exclusive feature developed for IoTCrawler is the resolution of nested filters made on top of NGSI-LD. Nested filters are equivalent to join clauses in traditional query languages (e.g., SPARQL), where multiple entity types can be returned or used as filters in a query. The recursive query resolution processor carefully passes through all the types used as filters or output fields and initiates the corresponding number of NGSI-LD requests. GraphQL queries designed and tested via GraphiQL (GUI) might be integrated into IoT applications and executed programmatically. Results are returned in machine-interpretable JSON format. Alongside the GraphQL-based search, the IoTCrawler is equipped with a rule-/pattern-based generator and mapping mechanism for generating filter conditions [7]. As a result, a state-based context model empowers GraphQL queries with context-based reasoning. The described Search Enabler’s search functionality is performed on top of the federated metadata infrastructure, which employs security and privacy-aware mechanisms.

The Search Enabler component offers a GraphQL interface, where search queries expressed in GraphQL are resolved via HTTP-requests over NGSI-LD interface. Since NGSI-LD allows to query only one type of entities per request, complex GraphQL queries (requesting more than one data type) require a corresponding number of NGSI-LD requests to be performed. The number and order of subsequent requests are prescribed by Search Enabler according to a structure of a GraphQL query. For example, a simple query of stream identifiers (streams{ id }) would be resolved by a single NGSI-LD request for entities with type iot-stream:IotStream (query #1). The extension of the query by the names of sensors (query #2) requires an additional resolution step: one NGSI-LD request for each sensor ID associated with the stream from the list of query #1. Further extension of query #2, e.g., by the names of properties observed by sensors, requires an additional resolution step: one NGSI-LD request for each property IDs associated with sensors. In case different sensors observe the same property, the Search Enabler avoids duplicating NGSI-LD requests.

For performance benchmarking, four different GraphQL queries have been selected. The difference between queries is in their complexity (requesting from 1 to 4 different entity types), which would require a different number of NGSI-LD requests to be performed. The expected number of NGSI-LD requests *N* depends on (1) a number of requested types *T* and if T>1, then on (2) a number of unique entities *R* of subsequent types referenced in the results set. More formally, it is described as follows:

(1) N=(T−1)∗R+1

**Figure 17:**
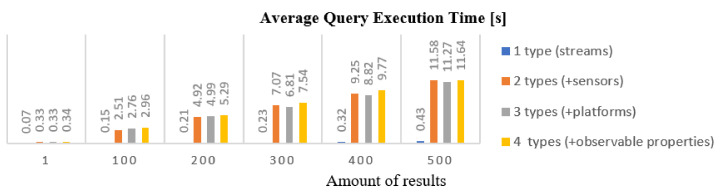
GraphQL query execution times.

**Figure 18:**
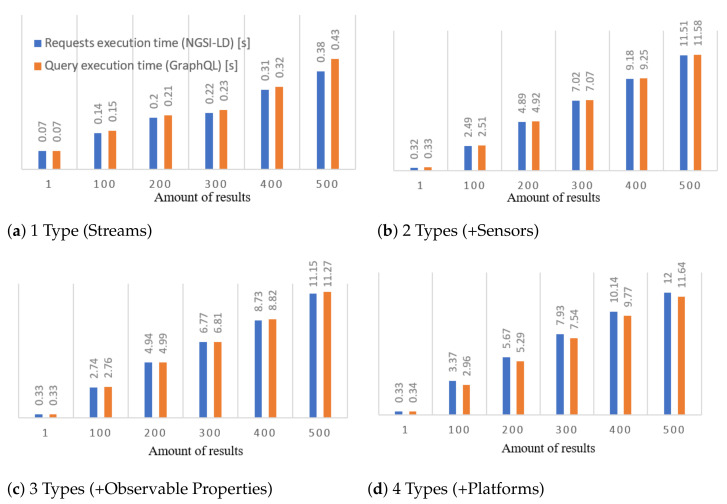
Requests execution time (Next Generation Service Interface for Linked Data (NGSI-LD)) vs. query execution time (GraphQL).

The caching mechanism avoids duplicating requests, so a real number of them might be significantly lower than was expected. During the experiments, we have measured the average GrahpQL query execution times and summarised the execution time of the corresponding NGSI-LD queries. Dependency on a number of results is demonstrated via limiting them within the range 1–500 with step size 100. Each experiment was repeated 10 times and the average times were calculated. In Figure 17, an average query execution time depending on number of results is demonstrated. Figure 18 represents a GraphQL query execution time against the summarised execution time of the corresponding NGSI-LD requests. From Figure 18d, it can be seen that GraphQL query execution goes faster than execution of the corresponding NGSI-LD requests. This can be explained by a particular query’s structure, where two types (observable properties and platforms) can be resolved in parallel. In the case of no parallel type resolutions (Figure 18a–c), the overhead of GraphQL engine is not higher than 0.2 s (1% of the overall query execution time). For complex queries with parallel type resolution, the overhead is mitigated at all. Experiments have been done using the NGSI-LD broker (Scorpio) running on Intel NUC i5-5250U with 8 GB of RAM. The Search Enabler and GraphQL client were running on a laptop Intel Core i7-5600U with 16GB of RAM, both were connected to a 1 GB/s local network.

The Search Enabler solves the machine-initiated search challenge (R-2) by providing programmatic interfaces (APIs), to which remote IoT applications can send search requests and get results back in an automated way.

### 5.4. Orchestrator

The Orchestrator component is targeted to be the mediating component for IoT applications, which are expected to be running outside the IoTCrawler platform, interacting with it via interfaces of the Orchestrator. The Orchestrator is an endpoint, which forwards all metadata requests to a Ranking component and subscription requests are forwarded directly to the MDR. At the same time, the Orchestrator provides its endpoint for receiving notifications coming from the MDR. Without it, applications would have to expose their own REST endpoint, which is often not possible (e.g., for apps running on mobile devices or in private networks). The Orchestrator mitigates that by providing its own endpoint (not exposed to the public) and redirecting all incoming notifications to a dedicated queue in a publicly available publish-subscribe service (Advanced Messaging and Queuing Protocol (AMQP)). It is enough for an IoT application to subscribe to a queue in the messaging service to get notified immediately. The described publish-subscribe mechanism also allows the setup to notify the IoT applications about stream failures detected by the monitoring component.

The Orchestrator implements the NGSI-LD interface and redirects incoming NGSI-LD requests to two components: MDR and Ranking. Entity subscription requests are analysed, modified (if required) and forwarded to MDR. The metadata/discovery requests are forwarded directly to the Ranking component, which allows it to rank the results of metadata requests according to a specified ranking criteria. As a result, the Orchestrator hides two IoTCrawler components under a single NGSI-LD interface—one of the interfaces used by IoTCrawler applications.

**Figure 19:**
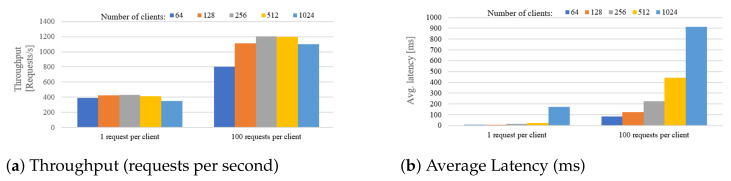
Orchestrator performance.

The evaluation of the Orchestrator component consists of measuring a dependency of performance characteristics (throughput and latency) on the number of parallel connections—IoT applications, running remotely. In this experiment, the Orchestrator component is working on top of Djane Broker—a lightweight NGSI-LD broker, which is less functional than Scorpio. The benchmarking process has been conducted using a single Intel Xeon machine (4 cores, 16 GB Ram). Each value was obtained by averaging the values of 10 repetitive experiments. Results can be seen in Figure 19. The number of parallel clients varied within the range 64–1024, where each client performed intensive and non-intensive workloads. For the non-intensive workload (1 request by each of 64–1024 parallel clients), the maximal average throughput is around 400 requests per second when the latency is less than 0.2 s. For intensive workloads (100 consecutive requests by each of 64–1024 clients), the maximal average throughput increases up to 1200 requests per second with the average latency increased to 1 s.

## 6. Application Domain Instantiation

This section presents two application examples of how IoTCrawler is being instantiated in real-world scenarios. Other scenarios for different domains are under development and will be part of a future publication.

### 6.1. Smart Home—Semantic Integration Focus

The target of the smart home use case was to understand the challenges which smart-home owners are facing when deploying and using their smart home devices. We have implemented an energy insight dashboard and tested it in a longitudinal study with end users in an early stage of the project. The energy insight dashboard was built with the objective to provide smart home users insights about their energy consumption and thereby to reduce their energy costs and carbon footprint. This was achieved by collecting energy measurements from smart plugs and other smart energy meters. The web-based application includes various aggregated and real-time views of the energy data as well as information about the usage frequencies of appliances attached to the smart plugs.

**Evaluation:** As part of IoTCrawler, we extended the dashboard to a public test bed running 24 h a week for almost a year. More than 60 homes and 3400 devices were connected during that period. Power users have more than two hundred devices connected to a smart home. Thus, we realised that managing these devices, which include knowing their locations, and for smart plugs, what kind of appliances are connected to which, created a considerable challenge for smart home owners. More importantly, the heterogeneity of devices with respect to their communication technologies, APIs and the gateways to which they are connected, makes it hard to develop smart home applications that run seamlessly with different vendors. As a response to tackling this challenge, we integrated an early version of an IoTCrawler feature for semantic annotation in which we used machine learning to detect device types, their locations and connected appliances in real-time [8]. We conducted a survey to validate the benefits of IoTCrawler features. Most of the respondents indicated that comparing and analysing energy usage is a benefit of the Energy Insights Dashboard (77%). On the second rank, respondents indicated that the automatic device detection feature is a benefit of the Energy Insights Dashboard (41%).

Further conversations with smart home owners and application developers have shown that IoTCrawler has the potential to be an effective IoT platform. For example, smart home users will be able to keep their data on their own hardware (located in private networks) and federate it into the IoTCrawler for processing by third-party analytical services. A Blockchain-based security mechanism (part of IoTCrawler) enables data owners to grant access to certain analytical services the similar way as a smart phone user grants access to certain mobile apps. Analytical service developers are considered responsible for managing their processing infrastructure and federating the processing results back to IoTCrawler. The core of IoTCrawler consists of the NGSI-LD standard together with a number of semantic ontologies, which makes data and metadata models more structured and understandable by independent service developers, which opens a potential for service compositions. As a result, raw data owners (smart-home users) will be authorised to access the intermediate (if needed) and final processing results calculated out of their data.

Encouraged by these findings, we further developed crawling and semantic annotation mechanisms to reduce time and effort when integrating smart home and other IoT and stream data into IoTCrawler. As IoTCrawler provides a common, semantic abstraction for finding and accessing the respective data streams, it becomes much easier to develop smart home applications. Consequently, we developed the “What’s happening at home” prototype that is fully implemented on top of the IoTCrawler infrastructure and interacts with the Orchestrator, Search Enabler, Ranking and Security components. The application detects users’ activities based on the energy consumption of appliances attached to smart plugs. Activities are modelled in terms of Home Activity ontology (http://sensormeasurement.appspot.com/ont/home/homeActivity accessed on 24 February 2021), which is partly described in one of the GraphQL schemas (https://github.com/IoTCrawler/Search-Enabler/blob/master/src/resources/schemas/homeActivity.graphqls accessed on 24 February 2021) used by the Search Enabler. The schema allows applications to filter households by type or location of detected activities (considering privacy policies). The developed application prototype demonstrates the separation between functionality and benefits from the granularity of the IoTCrawler data model by dealing with sensors and their streams.

### 6.2. Smart Parking—Security and Privacy Focus

Finding a free parking spot can be very cumbersome in populated cities with the collateral effects of having more vehicles circulating in the city, such as the increase in noise and pollution. In IoTCrawler, we provide a solution to alleviate this problem by offering a parking recommendation service, which allows the user to define the destination, time of arrival and the affordable walking distance. This solution takes advantage of IoTCrawler by gaining a way of representing the information homogeneously, allowing the new information to be introduced without any modification to our solution. More specifically, this solution uses Indexing and Ranking components to retrieve an ordered list of parking sites and parking meter information. Additionally, we allowed the data providers to specify different access policies, as an exercise for proving the security capabilities of our IoTCrawler platform, which the latter will affect the consumers in terms of the visibility of the information depending on the consumer’s attributes.

**Evaluation:** The SmartParking Most Valuable Product (MVP) is being tested in the City of Murcia, in the south-east of Spain. Previous to this solution, the City of Murcia had devoted efforts in research and development based on IoTCrawler, in order to incorporate and integrate promising solutions that would undertake the different challenges with respect to working with data from competing parking providers and regulated parking zones. The previous system was inspired by the participation in the CPAAS.IO project by the University of Murcia, where a solution for parking was devised, using technology derived from the FIWARE ecosystem: FogFlow. The parking solution based on FogFlow, utilised small “edge” devices that were to be installed in different parking locations, charged with the task of gathering data and performing local computations (such as aggregation or availability evaluation). This way, the system leveraged edge computing to enable quick and efficient data transfer, while relying on cloud resources for the heavy-lifting and edge workload-management centralisation. This solution already involved the use of NGSI interfaces for data access, which later on eased the transition to the next iteration, based on IoTCrawler. Some of the difficulties faced by the FogFlow approach were caused by some locations that already had online systems in place. They had special interfaces and connectors, which had to be developed in order to adapt the information and make it available to the rest of the system. In some cases, security and privacy were an issue, as providers wanted to be in control of what and was shared when with the system, and furthermore, how that information was to be accessed later by different parking solutions.

Those gaps have been successfully addressed by the IoTCrawler architecture, which provides a better and broader fit to the parking scenario, by introducing security through fine-detail policies that allow us to define how and whom is allowed to access or produce data. It also considers different ways of which data are to be incorporated into the system, be it directly from NGSI-LD enabled devices connecting to the parking system, through adaptation of other devices or even integrating entire existing systems through connectors and gateways. SmartParking leverages this security, providing a way to discriminate which end-users can access certain information. This way, a user could have permission to access specific parking alternatives. Although in our current implementation this functionality is only utilised by two fictitious users “Juan”, who has access to private parking, and “Pedro”, who has access to both parking and regulated parking zones. This functionality will allow us to introduce special user roles, such as medical professionals, who would have additional access to parking information for special private parking lots close to their hospital, or city officials that would have access to parking in official buildings, students having access to parking information in the city campus, etc. Furthermore, the security components of the platform would easily allow to define other flows of information coming from the end-users themselves, beyond the classical star ratings. This could mean the ability of claiming parking spaces, updating parking availability in zones with no (or poor) sensory information and it even opens up for future social/collaborative parking solutions, in which end-users can temporarily offer others their domestic parking lot while at work.

SmartParking, through IoTCrawler, copes with the diversity of data existing in the system, by using semantic technologies, such as those found in the semantic web. The extended usage in IoTCrawler of the NGSI-LD standard both for APIs and data modelling, allows the precise representation of information coming from different parking providers and allows for successful searches over highly diverse data. In a similar way to the previous FogFlow solution, which had a local scalability strategy based on the usage of edge devices as part of a distributed system, the IoTCrawler solution allows for the distribution of information through distributed MDRs, but it also provides a federation strategy that allows for broader and more diverse architectures, in which existing parking platforms can be integrated into IoTCrawler’s framework, enabling the federation with other parking systems. This federation capability, paired with the Indexing and Ranking components of IoTCrawler, as well as security components, allows for scaling beyond the local city to upper tiers, such as regional or national levels.

Finally, IoTCrawler integrates monitoring, fault-detection and fault-recovery mechanisms, providing useful data regarding the availability and reliability of the parking information contained in the system that can be directly used as part of the parking recommendation system with no further development needed. In short, the IoTCrawler approach for the SmartParking solution in Murcia, by far outperforms (feature-wise) the previous solution based in FogFlow, by accounting for the security aspect of data access, the diversity of data and the integration of existing solutions while allowing for greater scalability and flexibility to adapt and adopt new strategies and ideas, making it, in a way, future-proof.

## 7. Conclusions and Future Work

This paper presents the IoT search framework IoTCrawler, which allows for the search of data sources in the IoT. It features a domain-independent and layered design and provides solutions for crawling, indexing and searching of IoT data sources. Key enablers supporting the search process ensure privacy and security, scalability and reliability.

We started out the paper by presenting, several issues regarding an IoT search framework listed and analysed to build the basis for our requirements. These requirements have been successfully addressed by the IoTCrawler framework and its components. The loosely coupled components allow for different instantiations of the framework without blocking the search process. The scalability of the discovery and search enablers has also been evaluated to fulfil requirement R-1. With the adaptation of well-known ontologies and standards, an information model has been created to ensure a reliable basis for semantic annotation and context provision. This and the integration of standardised query interfaces enables the framework to be used for machine-initiated search queries R-2.

Requirement R-3 is addressed by designing the framework in a layered approach, which allows the discovery layer to work independently from the search layer. This enables crawling and discovery of new data sources, constantly semantically enriching and monitoring the data sources as well as building indices to speed up incoming search requests. In addition, it makes it possible to include existing solutions, it offers interoperability and overcomes data fragmentation and heterogeneity. As data sources in the IoT are often of private or restricted nature, security and privacy have to be considered R-4. Through the integration of an extensive security and privacy component, from design time on into the architecture of the framework, this requirement is successfully addressed.

To showcase the capabilities and applicability of IoTCrawler, two real-world instantations in different domains have been realised, featuring the search process in a smart home environment and the search in a Smart City use case. In future work, it is planned to enrol the IoTCrawler framework to further use cases covering other domains. This will bring “real” results and present how the framework could increase the benefits gained by the IoT.

## Figures and Tables

**Table 1:** Silhouette evaluation of Lagrangian representation using k-means.

Model/Data Set	Arrow Head	Lightning 7	Coffee	Ford A	Proximal
Raw Data k-means	0.47	0.12	0.33	0.05	0.46
Lagrangian k-means	0.67	0.57	0.69	0.56	0.62

**Table 2:** Results of Silhouette coefficient for the Aarhus data set.

Method	Silhouette Coefficient
PCA-Lagrangian + GMM	0.69
Raw data + GMM	0.46
Lagrangian scaling + GMM	0.45
PCA + GMM	0.39

## Data Availability

No new data were created in this study. Data sharing is not applicable to this article. Where available, source of data has been referenced in text.
